# Cerebral mechanism of Tuina analgesia in management of knee osteoarthritis using multimodal MRI: study protocol for a randomised controlled trial

**DOI:** 10.1186/s13063-022-06633-x

**Published:** 2022-08-19

**Authors:** Guangxin Guo, Yazhuo Kong, Qingguang Zhu, Zhiwei Wu, Shuaipan Zhang, Wuquan Sun, Yanbin Cheng, Min Fang

**Affiliations:** 1 Tuina Department, Yueyang Hospital of Integrated Traditional Chinese and Western Medicine, Shanghai University of Traditional Chinese Medicine, Shanghai, 200437 China; 2 Shanghai Municipal Hospital of Traditional Chinese Medicine, Shanghai University of Traditional Chinese Medicine, Shanghai, 200071 China; 3 Institute of Tuina, Shanghai Institute of Traditional Chinese Medicine, Shanghai, 200437 China; 4 School of Acupuncture-Moxibustion and Tuina, Shanghai University of Traditional Chinese Medicine, Shanghai, 201203 China; 5 CAS Key Laboratory of Mental Health, Institute of Psychology, Chinese Academy of Sciences, Beijing, 100101 China

**Keywords:** Knee osteoarthritis, Chronic pain, Tuina (Chinese massage), Analgesia mechanism, Multimodal MRI

## Abstract

**Background:**

The chronic pain of patients with knee osteoarthritis (KOA) seriously affects their quality of life and leads to heavy social and economic burden. As a nondrug therapy in Traditional Chinese Medicine (TCM), Tuina is generally recognised as safe and effective for reducing the chronic pain of KOA. However, the underlying central mechanisms of Tuina for improving the pain of KOA are not fully understood.

**Methods/design:**

This study will be a randomised controlled trial with a parallel-group design. A total of 60 eligible participants will be assigned to the Tuina group or healthcare education group (Education group) at 1:1 ratio using stratified randomisation with gender and age as factors. The interventions of both groups will last for 30 min per session and be conducted twice each week for 12 weeks. This study will primarily focus on pain evaluation assessed by detecting the changes in brain grey matter (GM) structure, white matter (WM) structure, and the cerebral functional connectivity (FC) elicited by Tuina treatment, e.g., thalamus, hippocampus, anterior cingulate gyrus, S1, insula, and periaqueductal grey subregions (PAG). The two groups of patients will be evaluated by clinical assessments and multimodal magnetic resonance imaging (MRI) to observe the alterations in the GM, WM, and FC of participants at the baseline and the end of 6 and 12 weeks’ treatment and still be evaluated by clinical assessments but not MRI for 48 weeks of follow-up. The visual analogue scale of current pain is the primary outcome. The Short-Form McGill Pain Questionnaire, Western Ontario and McMaster Universities Osteoarthritis Index, 36-Item Short Form Health Survey, Hamilton Depression Scale, and Hamilton Anxiety Scale will be used to evaluate the pain intensity, pain feeling, pain emotion, clinical symptoms, and quality of life, respectively. MRI assessments, clinical data evaluators, data managers, and statisticians will be blinded to the group allocation in the outcome evaluation procedure and data analysis to reduce the risk of bias. The repeated measures analysis of variance (2 groups × 6 time points ANOVA) will be used to analyse numerical variables of the clinical and neuroimaging data obtained in the study. *P*<0.05 will be the statistical significance level.

**Discussion:**

The results of this randomised controlled trial with clinical assessments and multimodal MRI will help reveal the influence of Tuina treatment on the potential morphological changes in cortical and subcortical brain structures, the white matter integrity, and the functional activities and connectivity of brain regions of patients with KOA, which may provide scientific evidence for the clinical application of Tuina in the management of KOA.

**Trial registration:**

Chinese Clinical Trial Registry ChiCTR2000037966. Registered on Sep. 8, 2020.

**Dissemination:**

The results will be published in peer-reviewed journals and disseminated through the study’s website, and conferences.

## Background

Knee osteoarthritis (KOA) is an important cause of knee pain and disability, causing serious personal and social burden all over the world [1, 2]. The total prevalence of KOA is about 17.0% from 2007 to 2015 in China [3, 4]. With the aging of the population, the prevalence of symptomatic KOA is increasing [1, 2, 5, 6]. The elderly population is estimated to reach 480 million by 2050, and the negative impact caused by KOA pain is bound to increase in severity [4]. A previous study has pointed out a 660 million increase in years of life lived with disability (YLDs) from 1990 to 2010, during which period the prevalence of KOA increased [7]. Many patients with KOA experience chronic pain [8, 9]. Pain is a common occurrence throughout the development and symptomatic phase of KOA, thereby seriously limiting the quality of life of patients. Symptomatic KOA is associated with increased risk of all-cause mortality amongst the residents in the rural areas [1, 10]. Pain, as a typical manifestation and the most common complaint of patients with KOA, has become a hot topic in clinical and scientific research [1].

The International Society for the Study of Pain redefines pain as an unpleasant sensory and emotional experience associated with, or resembling that associated with, actual or potential tissue damage [11]. KOA pain is also multidimensional; it is produced by the peripheral and central nervous system, and the regulatory mechanism is complex [12, 13]. The manifestations of KOA chronic pain are complex and varied, such as persistent pain or intermittent pain, with or without neuropathological components, with or without central sensitisation [14]. A previous study has suggested that pain may be associated with the changes in the functional activity and structures of the cortical and subcortical brain regions in the acute and chronic pain [15].

Chronic pain of KOA is complex [16]. The brain plays a key role in the occurrence and relief of chronic pain as studies have shown brain plasticity changes (e.g., grey matter (GM) structure, white matter (WM) structure, and brain functional activities) after chronic pain by using multimodal magnetic resonance imaging (MRI) [17]. Central sensitisation, as an important manifestation and pathological change of the central nervous system, plays an important role in the pathogenesis of knee arthritis [18, 19]. With the extensive application of brain MRI technology in pain research, investigators have attempted to map the brain regions involved in the central pathogenesis of chronic pain, and partly explored the central mechanism of Tuina analgaesia [20, 21]. The chronic and persistent pain as the defining symptom of KOA is closely related to pathological changes in cortical structure and subcortical brain nuclei [22]. Diffusion tensor imaging (DTI) provides the microstructure changes in brain WM by measuring the displacement of water molecules across brain tissue components. Fractional anisotropy (FA) obtained from DTI describes the degree of anisotropy of a diffusion process, which can show the treatment effects associated with changes in the specific area of WM fibre using voxelwise statistics on skeletonised WM tracts [23].

Tuina is recorded in the classic work of ancient Chinese medicine *Huangdi Neijing*, which plays an important role in the health of Chinese people for thousands of years. It takes advantage of the hands of the therapist to manipulate the surface of the patient’s body. The relaxation techniques in Tuina such as pressing and kneading exert significant analgaesic effect, improving mood, improving patients’ confidence in prevention and treatment, promoting rehabilitation, and reducing the use of health services [24, 25]. Tuina can also relieve the pathological fatigue of muscles, restore the physiological balance of muscles and bones [26], regulate the expression of TNF-α, MMP-13, integrin α1, and integrin β1 to inhibit the apoptosis of chondrocytes, promote the metabolism of cartilage extracellular matrix, and inhibit the degeneration of articular cartilage [27]. Moreover, numerous domestic and foreign studies have also shown that education is conducive to the treatment of KOA [28–34].

An education programme such as a self-management programme can provide knowledge and skills that will come in handy in helping patients improve the management of their condition. Self-management skills are essential for treatment and are strongly recommended in the guideline for osteoarthritis management [35]. For most KOA patients, the daily care and health management falls onto the patient himself and the family, who are not expected to have a regular interaction with the professional healthcare system [36]. Previous studies have confirmed the effectiveness of education programmes in reducing pain and disability [37, 38]. By comparing the MRI outcomes to those who undergo Tuina intervention, the unique brain area where Tuina intervention is activated can be determined. The findings will help to explain the difference between Tuina intervention and health education intervention from the cerebral mechanism and provide ideas to maximise the benefits of Tuina therapy.

Tuina is a promising treatment option for KOA [1]. It is based on the theory of meridians and acupoints and modern anatomy. Many studies have shown that Tuina is safe and effective for KOA in terms of pain, stiffness, physical function, quality of life, articular cartilage wear, ligament damage, and inflammatory response [39–42]. As regards the well-documented effects on pain, Tuina is recommended for the prevention and cure of KOA by the Chinese guideline and expert consensus [1, 43]. Under the guidance of the theory of TCM, doctors primarily use their hands to do the standard Tuina Manipulation on the patient’s body surface, which can dredge channels and collaterals, activate qi and blood circulation, etc. to relief pain and other symptoms [44]. Tuina treatment could affect brain plasticity such as structure and functional activity in the patients with chronic pain [20, 21, 45–47].

However, studies on the longitudinal effect and central analgaesic mechanism of Tuina in patients with KOA chronic pain are few [20, 48–50]. The current study aimed to (1) investigate the brain alternations in KOA chronic pain patients with combined clinical assessments and structural, DTI, and fMRI techniques; and (2) explore the longitudinal mechanism of brain plasticity changes after Tuina pain relief treatment for KOA patients compared with healthcare education treatment.

## Methods/design

### Participant recruitment

All patients with KOA will be recruited from the Tuina department of Yueyang Hospital of Integrated Traditional Chinese and Western medicine, Shanghai University of TCM. Recruitment methods will include posters, online advertisements, and leaflets. Written informed consents will be collected taken from all patients. A total of 60 patients with KOA chronic pain will be randomly assigned into a Tuina group and an Education group at 1:1 ratio.

This study’s protocol has been approved by the Ethics Committee of Yueyang Hospital of Integrated Traditional Chinese and Western Medicine, Shanghai University of TCM. The trial was also registered to the Chinese Clinical Trial Registry.

### Study design

This is a single-centre and parallel randomised controlled trial. A total of 60 participants diagnosed with KOA based on the American College of Rheumatology (ACR) criteria (2019 revised version) [51] will be recruited as eligible patients. They will be randomly allocated into 2 equal groups with 30 patients per group, including a Tuina group and an Education group. The interventions of both groups will last for 30 min and be performed twice each week. The treatment period will be 12 weeks, and the follow-up period will be 48 weeks.

Outcome measurements and MRI scans will be assessed at the baseline and at 6 and 12 weeks after treatment, followed by 48 weeks of clinical follow-up. The changes in clinical variables and cerebral activity of each group will be analysed after data collection.

**Fig. 1:**
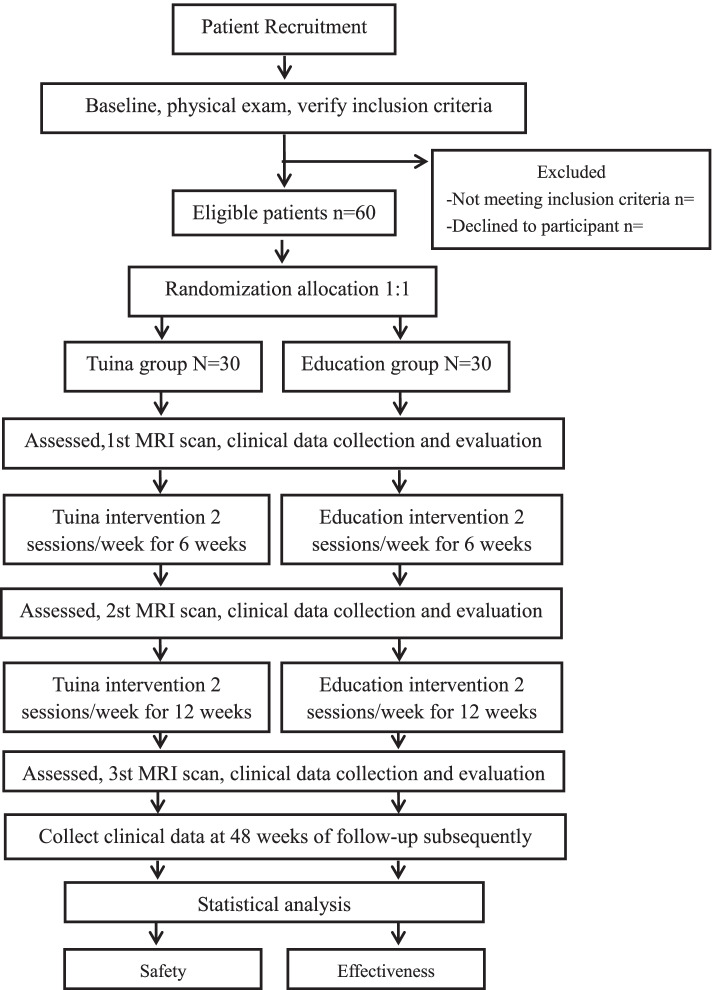
Flow chart of the trial. The present study is a randomized controlled trial using multimodal MRI. Sixty KOA patients will be included and randomized equally to two groups, a Tuina group and an Education group. For 30 patients in each group, this trial will include a 12-week treatment period. During the treatment, patients in the Tuina group will receive 24 sessions of Tuina treatments, while the Education group will receive health care education. Both the outcome assessments and MRI scan will be performed at 3 time points, namely the baseline, the end of the treatments at 6 weeks and 12 weeks. Only the clinical outcomes will be assessed during 48 weeks of follow-up subsequently. The central mechanism of Tuina in the treatment of KOA will be analyzed after data collection

**Fig. 2:**
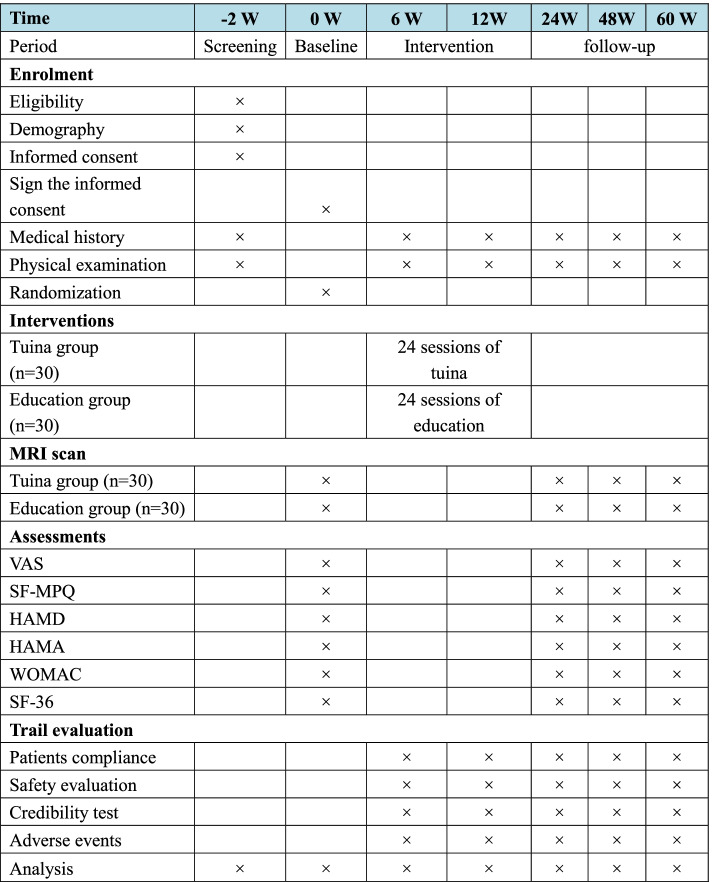
Study schedule for data collection. The informed consent and examination will be conducted after recruitment. Then, matched KOA patients will be randomized into two groups and receive treatment. Both clinical outcomes and MRI scans will be performed at 3 time points. Only clinical data will be collected at 48 weeks of follow-up subsequently. Adverse events will be recorded in the case report form at any time during the study. *VAS* visual analog scale, *SF-MPQ* the Short-Form McGill Pain Questionnaire, *HAMD* Hamilton Depression Scale, *HAMA* Hamilton anxiety scale, *WOMAC* the Western Ontario and McMaster Universities Osteoarthritis Index, *SF-36* the Short Form 36 Health Survey scale

Evaluators and statisticians will be blinded to the group allocation during the procedure of outcome evaluation and data analysis [52, 53]. The trial flowchart and study design are shown in Figs. 1 and 2, respectively.

### Inclusion criteria

1. Meet the diagnostic criteria for KOA set by the ACR in 2019 [51]
2. Are aged between 40 and 60 years, male or female, right-handed, with left knee pain
3. Have I–II degree knee joint radiological change on the Kellgren–Lawrence scale
4. Have an average knee pain score on a visual analogue scale (VAS) ≥ 3 (range from 0 to 10) in the past 3 months
5. Volunteer to take part in the study and sign the informed consent form
6. Can understand and follow the protocol.

Mainly to ensure the feasibility of the clinical study and the better baseline balance of the two groups, and because the incidence rate of KOA patients aged 40–60 is relatively high [54], so it is easier to recruit patients in the population aged 40–60. Since there are fewer patients with knee osteoarthritis under 40 years old, if patients under the age of 40 are recruited, the baseline of the two groups may not be easy to reach balance, and the age factor will also increase the difficulty of exploring the brain mechanism of knee pain. The patients older than 60 years old are more likely to develop pathological manifestations such as brain diseases and pain in other parts of the body [55], which also makes it more difficult to study and explore the brain mechanism of knee pain. According to our clinical observation, we found that there are more right-handed patients in the outpatient department of our hospital. Recruiting right-handed patients can avoid the influence of left-handed factors on the study results. Moreover, when patients with knee osteoarthritis have left knee pain, the brain signals detected are generally reflected in the patient’s right brain centre. If patients with right knee pain are recruited simultaneously, brain signals will also be detected in the left brain centre. However, this situation is not conducive to the combined calculation and statistical analysis of brain image data, nor is it conducive to revealing the precise range of brain areas of knee pain. Therefore, we will only recruit KOA patients who are aged between 40 and 60 years, male or female, right-handed, with left knee pain.

### Exclusion criteria

1. Are taking analgaesics or anaesthetics in the past 1 month that may influence brain-imaging outcomes
2. Have received any other treatment in the past 1 month, such as exercising therapy, pharmacological intervention, or operation
3. Are pregnant or lactating women
4. Are suffering from mental disease, neurological disease, infectious disease, gastrointestinal disease, cardiovascular and cerebrovascular disease, immunologic disease, respiratory disease or kidney disease, any other chronic pain symptoms, or have a history of brain injury with loss of consciousness
5. Are diagnosed as cancer, tuberculosis, rheumatism or rheumatoid arthritis, gout, or joint trauma
6. Have MRI contraindications such as claustrophobia, cardiac pacemaker, defibrillator, heart stent, or intrauterine device
7. Have skin lesion around the knee joint

### Suspension criteria

1. Are unsuitable to continue to participate in the study owing to adverse events or serious adverse events during the trial
2. Are unsuitable to continue to participate in the study because of serious deterioration of disease or some complications and special physiological changes
3. Uses concomitant medication during the 12-week treatment period is a suspension criteria. Non-compliance to concomitant interventions will be monitored by daily dairies and WeChat queries.

### Dropout criteria

1. Withdrew from the study
2. Loss of contact

### Sample size

Sample size is required to compare two group mean values. The calculation method of bilateral equality of two samples is adopted [56] for two groups of A = Tuina group and B = Education group. Suppose H0: μA−μB=0, H1: μA−μB≠0. According to previous relevant research and pre-experiment results [39, 57], we expect the VAS significant score difference between and after 12 weeks of Tuina as μA = 3, and the VAS score difference between and after healthcare education intervention as μB = 0.7, assuming that the standard deviation *σ* of the two groups is equal, i.e., *σ*=2.1. If we set *α*=0.05 as the type I error, *β*=0.10 as the type II error, and 1−*β*=0.90 as the power, the final result is that we need at least 25 samples in each group. For the primary outcome, VAS will be assessed at baseline and at the end of 6 and 12 weeks of treatment, followed by 12, 36, and 48 weeks of follow-up. The statistical significance is defined as *P* < 0.05, and the 95% confidence interval will be reported.

Many MRI studies have reported statistical significance with 12 to 21 patients per group [23, 58–60]. To have a powerful and repeatable statistical effect, we require 25 patients per group in this trial. Considering a 20% dropout rate and the possible excessive head motion during scanning, it will include 30 KOA participants in each group. In summary, we plan to enroll 60 participants, and each group will undergo MRI scans to investigate different central mechanisms between Tuina and healthcare education treatments.

### Randomisation

A total of 60 eligible participants will be assigned to the Tuina or Education groups at 1:1 ratio using stratified randomisation with gender and age as factors in this research. Stratified randomisation is achieved by performing a separate randomisation procedure within each of two subsets of participants (gender and age). First, participants will be grouped by gender. Then, male and female participants will be grouped by age. After grouping, there will be four subgroups: (1) men aged < 50, (2) men aged ≥ 50, (3) women aged < 50, and (4) women aged ≥ 50. In these four subgroups, the subjects will be randomly divided into the Tuina group and Education group. Finally, all Tuina groups and Education groups are merged to form a new Tuina group and Education group. This step ensures good balance amongst participant characteristics in each group and close balance amongst the numbers of participants receiving each intervention within each stratum. The created random number lists will be generated by a random number generator (IBM SPSS Statistics version 21.0 software; IBM Corp., Armonk, NY, USA) and will be sent to the therapist with sequentially numbered, opaque, and sealed envelopes by an independent assistant. The therapist will open random-allocation envelopes and allocate the participants accordingly to the Tuina or Education group.

### Blinding

Participants and therapists will not be blinded to treatment allocation, because of the features of the Tuina treatment and the education intervention. For participants, their subjective consciousness will affect the outcomes, which is unavoidable. Nevertheless, the evaluators, data managers, and statisticians will be blinded to the group allocation in the outcome evaluation procedure and data analysis throughout the entire procedure to reduce the risk of bias. The data will not be accessible to the evaluators until the collection is finished.

### Interventions

The participants will receive a total of 24 treatments in 12 weeks. The interventionists, who hold a PhD, must have at least 10 years of experience with Tuina treatment and be skillful in healthcare education for the participants. They are also required to have passed a clinical test to ensure consistency of the trial. The clinical test primarily includes the time of Tuina, the positioning of the treatment site, and the application of specific Tuina manipulation types. The standard operating procedures primarily include Tuina’s time, treatment site, and manipulation category, as well as the time and content of health education. In the process of Tuina or Education intervention, the two groups will not allow any concomitant medications or other interventions. However, concomitant medications or other interventions can be used during subsequent follow-up, and the treatment will be recorded in detail.

### Tuina group

The selected acupoints for the Tuina treatment include EX-LE02 (heding), EX-LE04 (neixiyan), EX-LE05 (xiyan), GB34 (yanglingquan), SP10 (xuehai), ST34 (liangqiu), ST32 (futu), BL40 (weizhong), BL57 (chengshan), GB31 (fengshi), and Ashi acupoints around the knee joint. An ashi point is a temporary acupoint where sensation of acid, distension, and pain exist, which can appear anywhere in knee joints [61].

Referring to previous studies [44, 62–64], the Tuina procedures are as follows. First, the patients will be in a supine position. The doctor stands on the side of the patient. The Pressing and Kneading Manipulation of Tuina is applied to the anterior acupoints around the affected knee joint for 10 to 15 min by using the thumb to achieve Deqi sensation, which is commonly regarded as an indicator of manipulation efficacy [65, 66]. Second, the patients will be in a prone position. Then, the physician will perform the same manipulation on the patient’s posterior acupoints around the affected knee joint for 10 to 15 min. The purpose is to relax sinews and activate collaterals according to the theory of TCM. Every patient will receive 30 min of manipulation per treatment with two treatments per week for 12 weeks.

### Education group

The Osteoarthritis Research Society International (OARSI) and the European Society for Clinical and Economic Aspects of Osteoporosis, Osteoarthritis and Musculoskeletal Diseases (ESCEO) updated the guidelines on KOA in 2019 [67]. In the guidelines, both organisations recognised the efficacy of health education in the treatment of KOA. Therefore, in this experiment, we will use health education to implement interventions in a group of subjects.

The education intervention will be given to the participants with two sessions per week. The first health education session is a group meeting using a PowerPoint presentation, which will last 30 min. An educational booklet and a video will be provided to the patients to accomplish the home-based online e-learning module. The later sessions will be conducted as one-to-one communication focused on personal needs to guide patients to implement the key education in their lives. The programme includes two broad components: (1) introduce key concepts of pain biology, and (2) present specification of recovery.

Each patient will be given an introduction about the underlying mechanisms, predisposing factors, and prognosis of KOA. Pain will be explained as the conscious part of the response, which can be influenced by many factors as a protective output [68]. Interventionists will formulate a specification of daily routines for patients, for example, keeping the knees warm, keeping a healthy diet and preventing obesity [69], maintaining beneficial posture and abandoning bad habitual posture [70], and avoiding too much work.

### Concomitant medications and other interventions

Patients with KOA will be instructed not to take any caffeine (e.g., tea and coffee), as well as other medications, weight-loss techniques, or physical activity to avoid confounding interference for the analgaesic effect of the intervention in the two groups. In cases of severe knee pain, ibuprofen (300 mg per capsule with sustained release) will be allowed as rescue medication and should be recorded on a knee-pain diary. After the end of the study, patients will be taught weight-loss techniques and physical activity. Patients are also asked to record the name, dose, date, frequency, and exact time of the medications used, as well as to report to the researcher if they take any medications during the study. Patient adherence will be maintained and monitored by good communication between the therapist and patient and by constant reminders.

### Safety evaluation

The participants’ conditions will be carefully monitored by therapists during the process. If adverse events occur, participants with adverse events will stop participating in the trial. Immediate medical care will be provided to any participant experiencing adverse reactions that can lead to hospitalisation, for example fracture or coma. The details will be recorded in the case report forms (CRFs). The ethics committee will conduct an interim analysis and make a decision, which will be determined according to the severity of the adverse events of the participants. For example, conditions that lead to hospitalisation or even life-threatening are severer, whereas conditions such as muscle pain that can heal on their own are milder. Reports will be made to the ethics committee with statements and detailed reasons for trial suspension. The efficacy and safety of the intervention will be evaluated after 6 and 12 weeks of intervention and 48 weeks of subsequent follow-up. Evaluators can also be reformed with the clinical symptoms and AEs whenever the patients meet something important. After an adverse event is determined to be related to the trial plan, the research team will provide treatment costs and corresponding financial compensation for the damage inflicted by the trial plan.

### MRI data acquisition

MRI data will be acquired using a 3.0-T magnetic resonance scanner (SIEMENS MAGNETOM Verio syngo MR B17, Germany) with a 32-channel phase-array head coil at the Medical Imaging Department of Yueyang Hospital of Traditional Chinese and Western Medicine, Shanghai University of TCM.

On the pre-MRI evaluation procedure, the scanner will instruct the subject to rest for 10 min, relax, and keep calm. Throughout the entire scan, participants are asked to close their eyes, wear earplugs, keep their heads still throughout the scan, stay relaxed and awake, and not think of anything in particular.

Prior to the blood-oxygen-level-independent (BOLD) resting-state functional images, a high-resolution T1 image for each subject will be acquired with spin echo (SE) sequence, transverse sagittal scan, flip angle = 9°, pulse repetition time (TR) = 1900 ms, echo time (TE) = 2.93 ms, field of view (FOV) = 256×256 mm^2^, number of slices = 160, and slice thickness = 1 mm.

DTI will be measured with the axial DTI mapping sequences, TR = 10,000 ms, TE = 89 ms, matrix = 240 × 240, slice thickness = 2 mm, B-value1 = 0 s/mm^2^, B-value2 = 1000 s/mm^2^, and 30 directions.

The BOLD resting-state functional images will be obtained with an echo-planar imaging sequence as follows: coronal axial scan, 33 slices, 4 mm thickness, TE = 30 ms, TR = 2000 ms, FOV = 220 × 220 mm^2^, voxel size = 3.4 × 3.4 × 4.0 mm^3^, FA=90°, scanning time = 8 min 8 s, 240 volumes in total.

On the post-MRI evaluation procedure, the subjects will be instructed to rest for 5 min. They may leave if there is no uncomfortable feeling.

The MRI outcomes include the following: structure–GM density, cortical thickness, subcortical nuclei volumes; diffusion–FA and MD of WM integrity; fMRI–functional connectivity (FC).

The 3D T1 structure data analysis will be performed with FSL tools (FMRIB Software Library) [71]. SIENAX (part of FSL 5.0) [72] will be used to obtain the volumes of neocortical GM, total GM, and WM. The normalised volumes of subcortical regions such as hippocampus and thalamus will be estimated from FMRIB’s integrated registration and segmentation tool (part of FSL 5.0, FMRIB Software Library) [73]. The cortical thickness at each vertex will be obtained using FreeSurfer (http://surfer.nmr.mgh.harvard.edu). DTI data-processing pipeline will follow the diffusion toolbox in FSL to obtain FA and MD images, and voxelwise tract-based spatial statistics method will be applied to examine FA/MD changes. For the resting-state fMRI data, preprocessing and functional connectivity analyses will be performed with SPM12 software (SPM12, Wellcome Department of Imaging Neuroscience, London, UK; http://www.fil.ion.ucl.ac.uk/spm/) by using MATLAB 2013b (Mathworks, Inc., Natick, MA, USA).

### Outcome measurements

The clinical outcome will be measured by six self-report questionnaires at baseline and the end of 6 and 12 weeks of treatment, followed by 12, 36, and 48 weeks of follow-up. The responses can reflect the degree of pain, sensation, emotion, and quality of life.

#### Primary outcome measurement

The primary outcome measurement is VAS [74]. VAS is used to assess the current degree of pain. VAS is a 10-point scale selected to quantitatively measure the level of KOA pain during the study, for which 0 means no pain and 10 means unbearable pain. The VAS mean scores will be assessed by repeated longitudinal analysis. The single timepoint of the primary interest for the comparison is week 12.

#### Secondary outcome measurements

The secondary outcomes are Short-Form McGill Pain Questionnaire (SF-MPQ) [75], Western Ontario and McMaster Universities Osteoarthritis Index (WOMAC) [76], and the 36-Item Short Form Health Survey (SF-36) [77]. SF-MPQ assesses the intensity and component of knee pain, WOMAC will be used to assess the intensity of KOA symptoms in multiple dimensions. SF-36 will be used to measure the Quality of life (QoL) of the participants. HAMA will be used to measure the level of anxiety of the participants. HAMD will be used to measure the depression level of the participants.

SF-MPQ is used to evaluate the pain, emotion, and psychological status of patients with KOA. It primarily comprises a 15-descriptor (11 sensory and 4 affective) rating on an intensity scale as follows: 0 = none, 1 = mild, 2 = moderate, and 3 = severe. A higher total score indicates more severe pain. The WOMAC is a self-administered questionnaire comprising 24 items divided into three subscales: pain (5 items), stiffness (2 items), and physical function (17 items). These measurements will be used to evaluate the symptom and quality of life improvement. A repeated longitudinal analysis will be used to evaluate the SF-MPQ mean scores.

Furthermore, to investigate the influence of emotional state on the brain activity, HAMD [78] and HAMA [79] will be used.

Most items of the HAMD adopt the 5-level scoring method of 0–4 points. The standard of each level as follows: 0 = none; 1 = mild; 2 = moderate; 3 = severe; 4 = extremely severe. A few items adopt the 3-level scoring method of 0–2 points, and the grading standard as follows: 0 = none; 1 = mild to moderate; 2 = severe. The total score of HAMD is 78. A total score less than 8 means normal. A total score of 8–20 means possible mild depression. A total score of 20–35 means possible depression. A total score greater than 35 means severe depression. A repeated longitudinal analysis will be used to evaluate the HAMD mean scores.

The total score of HAMA can better reflect the severity of anxiety symptoms. The total score can be used to evaluate the severity of anxiety symptoms and the effect of various drugs and psychological intervention in patients with anxiety and depression. According to the data provided by the China scale collaboration group, a total score ≥29 means possible serious anxiety, ≥21 means possible obvious anxiety, ≥14 means anxiety, ≥7 indicates possible anxiety, and <7 means no anxiety symptoms. A repeated longitudinal analysis will also be used to evaluate the HAMA mean scores.

### Data collection and monitoring

The screeners will collect data on the baseline characteristics when the patients are recruited. CRFs include observation and scanning time points, outcome measures, adverse events, and safety evaluations. Doctors are blinded to evaluate various clinical pain indicators and fill-in relevant information timely and accurately according to the CRF requirement. Only outcome assessors can access the CRFs and perform double-data entry. Then, two data administrators, who are beyond the research team and blinded to group allocation, will independently receive the completed CRF and enter them into an Excel database (Microsoft, Redmond, WA, USA). They are required to have completed rigorous training for the data monitoring. Then, they will enter the real-time data into the Chinese Clinical Trial Registration Center, in which the electronic data-management system will be used to track and monitor the test data in real-time in the Department of Science and Technology in Yueyang Hospital. During the test, data administrators can access and manage data. After the test, all researchers will have access to the data, and non-researchers will not have access.

### Statistical analyses

Clinical data will be analysed through the intention-to-treat assumption. For all outcomes, we use longitudinal models where participant outcomes and treatments are collected at a multiple follow-up times. Moreover, participants who meet the suspension criteria will be removed from the trial, and the modified intention-to-treat analysis will be adopted. For the missing data, several sensitivity analyses will be conducted to evaluate their effects on the results of the experiment. All analyses were performed with SPSS 21.0 statistical software (IBM, Armonk, NY, USA) by statisticians who are blinded to the group allocation.

The baseline characteristics will be expressed with descriptive statistics for the two groups, which are reported as the mean ± standard deviation. A Kolmogorov–Smirnov test with Lilliefors correction will be used to analyse all quantitative variables to determine whether they follow a normal distribution. Parametric statistics (Tukey test) or nonparametric statistics (Wilcoxon rank-sum test) will be used for the within- and between-group analyses in accordance with the results of the homogeneity and normality analyses. When the initial homogeneity and normality of data distribution are found, repeated-measure ANOVA will be implemented, or the Friedman test and Kruskal–Wallis test will be used when initial homogeneity but non-normality of data distribution is found, or only linear mixed model will be adjusted for age and gender if the initial homogeneity is not found. Adverse events in each group will be documented as percentage for safety assessments by using the chi-square test or Fisher’s exact test. Statistical significance is defined as *P* < 0.05, and the 95% confidence interval will be reported.

### Quality control

During the processing of the trial, quality control will be conducted with the management of the steering committee. To ensure the consistency of methods, professional trial method and regular monitoring technique should be trained before the researchers participate in the trial. If the study protocol is modified or corrected, the steering committee and ethics committee should be informed. The steering committee and ethics committee will ensure that the trial process works as planned, such as monitoring recruitment, potential harms, Tuina intervention, Education intervention, and data quality.

## Discussion

Chronic pain in KOA often seriously affects the patients’ quality of life. Alleviating joint pain is an important part of KOA treatment recommendations in the international OARSI guidelines (2014) [80] and the Chinese Osteoarthritis Diagnosis and Treatment Guidelines (2018) [1]. Chronic pain of KOA is not completely consistent with the clinical imaging performance of the knee joint, meaning that the results of knee X-rays should not be used in isolation when assessing individual patients with knee pain [81]. KOA also reportedly has a high probability of pain after successful operation [82]. The reason may be the key role of the brain as the integration centre of neural signals [83]. This study will provide a more powerful evidence-based proof for Tuina therapy in KOA patients with chronic pain through the possible study outcomes and benefits of longitudinal data.

Tuina belongs to the key noninvasive and nondrug therapies of TCM with distinctive characteristics [84, 85], which is used for pain relief in KOA for more than a thousand years in China and some other surrounding countries. Education, as a nondrug therapy for KOA recommended by both OARSI and ESCEO, is important for the treatment of patients with KOA [67], which can improve the living habits and relieve the pain of KOA. Although the thalamus is an important part of the pain matrix, previous studies have also found abnormal thalamus structure in patients with KOA [86]. However, the regulatory mechanism of thalamus and related brain regions involved in Tuina analgaesia remain unclear, especially the relationships amongst different brain tissues and the relationship between different brain tissues and clinical behavioural indicators, thereby limiting the promotion and application of Tuina. Therefore, fully exploiting the advantages of Tuina and evaluating the analgaesic effect and central mechanism are necessary to better alleviate the pain of KOA.

Multimodal MRI is a cutting-edge technology in the study of pain brain-network mapping and analgaesia mechanism. It can explore the mechanism of pain and evaluate and predict pain. It is extensively used in brain science and pain research. Brain MRI is one of the most commonly used neuroimaging technologies in the study of central mechanism given its advantages of its high-quality spatial resolution, no radiation, rapid imaging velocity, non-invasion, and affordable price [86–89]. The pathogenesis of chronic pain and persistent pain of KOA are closely related to the subcortical structures and morphological parameters of subcortical nucleus [22]. DTI can quantitatively measure diffusion tensor and FA to examine the microstructure and macrostructure of WM in vivo [90, 91]. fMRI is widely used in the clinical and theoretical studies of Tuina analgaesia for KOA [92], cervical spondylosis [45, 93], lumbar disc herniation [94, 95], low back pain [21], etc. [62, 96–98]. fMRI can also be used to reveal the central mechanism of Tuina analgaesia, which is related to the functional changes in amygdala, hypothalamus, nucleus accumbens, hippocampus, cingulate gyrus, and other areas [92, 96]. As a main type of fMRI, resting-state fMRI has the advantage of providing more comprehensive information than task-related fMRI on the functional architecture of the brain [99]. The cross-study on this frontier technology and Tuina can better explain the mechanism of analgaesia and solve the pain problem.

This clinical trial will study the multidimensional evaluation of KOA pain and evaluate the physical function, quality of life, mental status (the level of anxiety and depression), and influence of emotional state on brain activity. We will use a combination of clinical behaviour and multimodal MRI indicators such as brain-function indicators, brain-structure indicators (FA, MD, and GMV), and brain-network properties to perform confirmatory and interactive Tuina analgaesia research, which can fill the knowledge gaps in brain pivot analgaesia research in Tuina discipline in this field.

Furthermore, owing to the great difference between humans and animals, as well as the particularity of Tuina manipulation, verifying the central regulatory mechanism of Tuina intervention on KOA analgaesia in animal experiments is difficult because of too many uncontrollable factors. The application of multimodal MRI to Tuina discipline can clearly explore the brain central characteristics of KOA pain only in clinical trial, as well as comprehensively and deeply analyse the central analgaesic regulation mechanism of KOA patients with chronic pain after Tuina, which is a new trend in the future research of Tuina. We can also provide a reference experimental paradigm for the cross-study on the analgaesic effect of TCM nondrug therapy.

## Study limitations

This research has an inevitable limitation associated with the difficulty in controlling the methodology of blinding during the Tuina and healthcare education intervention, which cannot be blinded for the participants and therapists. Patients can know their group and anticipate the effect of the treatment in advance, which may affect their outcomes. For a study involving complex, multicomponent mind–body therapy, identifying a feasible, useful, and valid sham comparison group remains challenging, with no well-accepted solution.

## Trial status

This trial is recruiting patients now. Participant recruitment started in Sep. 2020 and is expected to end in Dec. 2022. The version of this protocol is the 1st version and the completion time is 8 Sep. 2020.

## Abbreviations

KOA: Knee osteoarthritis
VAS: The visual analogue scale
SF-MPQ: The Short-Form McGill Pain Questionnaire
HAMD: The Hamilton Depression Scale
HAMA: The Hamilton anxiety scale
WOMAC: The Western Ontario and McMaster Universities
SF-36: The Short From 36 Survey Questionnaire
